# Decomposing differences in the chronic disease condition between rural and urban older adults in China: a cross-sectional analysis

**DOI:** 10.3389/fpubh.2023.1298657

**Published:** 2024-01-05

**Authors:** Jian Zhang, Yuan Zhang

**Affiliations:** ^1^School of Public Administration, Xi'an University of Architecture and Technology, Xi'an, China; ^2^School of Law and Public Administration, China Three Gorges University, Yichang, China

**Keywords:** chronic diseases, differences, rural and urban older adults, Fairlie's decomposition, CHARLS

## Abstract

**Background:**

With the increasing in aging in China, there has been an increase in older adults suffering from chronic diseases. However, little is known about the differences in chronic disease conditions between rural and urban older adults. The objective of this study is to identify chronic disease conditions and investigate the factors that cause differences in chronic disease conditions between urban and rural older adults.

**Methods:**

The data are from the fourth wave of the China Health and Retirement Longitudinal Study. The coarsened exact matching (CEM) method was used to reduce the biases for a comparative study. After the CEM method, this study included 5,927 participants aged 60 and above. Chronic disease condition was used as the indicator to measure the health of older adults. Specifically, Fairlie's decomposition analysis was carried out to discover the differences in chronic disease conditions between urban and rural older adults.

**Results:**

The study showed that the proportion of those suffering from chronic diseases was significantly higher among urban older adults (51.26%) than rural older adults (46.56%). In those suffering from chronic diseases, there were significant differences in gender, education level, minorities, religiosities, duration of sleep, drinking alcohol, social activity, insurance, and socioeconomic status between rural and urban older adults, while in those not suffering from chronic diseases, there were significant differences in age, education level, marital status, drinking alcohol, social activity, insurance, region, and socioeconomic status between rural and urban older adults. For rural older adults, those who were widowers [Odds ratios (OR): 1.267], who drink alcohol (OR: 1.421), and having government medical insurance (OR: 4.869) had higher odds of having chronic diseases. However, those who were in high school and above (OR: 0.802), reporting a duration of sleep of 4–8 h (OR: 0.745) or above 8 h (OR: 0.649), having social activity (OR: 0.778), and having the most affluent socioeconomic status (OR: 0.778) had lower odds of having chronic diseases. As for urban older adults, those who were aged 65–74 years (OR: 1.246) and had government medical insurance (OR: 2.362) had higher odds of having chronic diseases. Fairlie's decomposition analysis indicated that 23.57% of the differences in chronic diseases conditions could be traced to duration of sleep, drinking alcohol, social activity, and region.

**Conclusion:**

This study illustrated that the proportion of chronic diseases was higher among urban older adults than rural older adults. Considering duration of sleep, drinking alcohol, region, social activity, and region, the study demonstrated health differences between urban and rural older adults and provided evidence for policy-making to narrow the health gap between urban and rural areas.

## 1 Introduction

The global aging population is rapidly increasing. The percentage of the world's population of people aged 60 years and above will almost double from 2015 to 2050 (1). In China, the number of adults aged 60 years and above had grown to 264 million by the end of 2020, representing 18.7% of the total population (2). By the year 2050, China's population of people aged 60 years and above is predicted to reach 498 million (3). As aging progresses, suffering from chronic diseases has become a worldwide public health issue and is a major health concern in low- and middle-income countries (4–6).

According to the World Health Organization (WHO), chronic diseases are afflictions that persist for an extensive duration and frequently advance progressively, which include cardiovascular disease, cancer, chronic respiratory disease, and diabetes (7). High incidence, prevalence, disability, mortality, and medical costs are characteristics of suffering from chronic diseases, which significantly affect the quality of life of residents (8). According to the WHO, in *Focusing on non-communicable diseases*, chronic diseases have become one of the main causes of death worldwide and have been one of the most significant health issues worldwide (9). Chronic diseases led to 88.5% of all deaths in China in 2019. Even worse, due to population aging, advanced urbanization, industrialization, and behavioral risks, chronic diseases will persistently escalate in China (10). Risk factors for chronic diseases include smoking, insufficient physical activity, poor diet, hazardous alcohol consumption, and so on. The management of chronic diseases is not only limited to clinical intervention but also influenced by lifestyle and societal structures. It is a complex system that needs coordination, so the United Nations has identified chronic diseases as one of the major global challenges (11).

The differences in health status between urban and rural older adults are important aspects of older adults' health studies. For example, Saha et al. (12) found that urban older adults had better health status than rural older adults in India. Moss et al. (13) found that the burden of chronic diseases was higher among rural older adults than among urban older adults in the mid-Atlantic United States. In South Africa, Peltzer et al. (14) found that urban older adults had a higher prevalence of diabetes, edentulism, and cognitive functioning than rural older adults. Moreover, Norris et al. (15) found that urban–rural health disparities existed in 21 African countries. These studies presented empirical evidence to improve the health disparity between urban and rural older adults.

Chronic diseases are prevalent diseases associated with biological, psychological, and social factors (16). Studies have shown that personal and behavioral characteristics could lead to chronic diseases, and individuals' health is also affected by complex social environments (17). For example, different marital status and place of residence often lead to differences in personal health (18). Grossman (19) put forward the concept of health capital in 1972, showing that factors influencing residents' health included personal attributes, social backing, financial standing, and lifestyle. Wilkinson and Pickett (20) found that the economic status of individuals in developed countries was more likely to be a determining factor in their health. Furthermore, Bassuk et al. (21) found that regional socioeconomic situations influenced the correlation between personal wealth and health.

Demographic factors, lifestyle, and environmental elements predominantly influence chronic disease incidence among older adults (22–24). For example, Gaballa et al. (25) found that premature mortality from chronic diseases, including hypertension, diabetes, and coronary heart disease, escalated with age. Ahmed et al.'s (26) study showed that older adults with higher levels of education had a higher risk of developing hyperlipidemia. Irigaray et al. (27) found that lifestyle was the key risk factor of suffering from chronic diseases, including smoking, alcoholism, bad eating habits, lack of physical activity, and so on. The WHO pointed out that smoking heavily increased global chronic disease prevalence. To date, almost all of the substances in tobacco are harmful to the human body, and more than 40 carcinogens have been identified (28). Makimoto and Higuchi (29) found that heavy alcohol consumption was a major determinant of liver cancer and death due to liver cancer in Japan. The WHO promoted physical activity that could reduce the prevalence of chronic diseases (30). Numerous studies have demonstrated a correlation between socioeconomic status and chronic disease prevalence (31, 32). A study involving individuals aged 60 years and above indicated that economic status significantly influenced the chronic disease prevalence in the older demographic group (31). Concerning research methodology, most studies examined the differences in chronic disease conditions between rural and urban older adults using multiple regression. However, these studies could not explain the rural-urban health differences. To solve the issues contributing to health differences between urban and rural older adults with chronic conditions, decomposition analysis is necessary. Therefore, this study aimed to decompose the differences in chronic disease conditions among rural and urban older adults in China, identifying contributing factors. This study may enrich the understanding of health among older adults in China, and the findings can also provide a reference for narrowing the health gap.

## 2 Methods

### 2.1 Data

The fourth wave survey data from the China Health and Retirement Longitudinal Study (CHARLS, wave 4), which was released in 2020, was used. CHARLS contains micro-data reflecting Chinese households and individuals aged 45 years and above, providing a chance to explore China's population aging conundrum and invigorating multidisciplinary aging studies. The CHARLS baseline survey ensured nationwide representation by covering 150 countries/districts and 450 villages/urban communities, involving 17,708 individuals in 10,257 households representing older adults in China. A multi-tiered probability proportional to size sampling (PPS) and random selection method was utilized, stratified by urban district and rural county per capita GDP (33). The samples can be trackable every 2 or 3 years, and post-survey data could be made available to the academic community annually.

### 2.2 Chronic disease condition measurement

Chronic disease condition among older adults was measured through 14 questions related to chronic diseases. The questions are in the attached materials. For these 14 questions, respondents answered “yes” or “no.” Answering “no” was defined as “not suffering from chronic diseases” and answering at least one “yes” was defined as “suffering from chronic diseases.”

### 2.3 Description of variables

Numerous variables were considered in CHARLS based on prior empirical pieces of research (34–38). Variables related to chronic disease conditions between rural and urban older adults are shown in Table 1.

**Table 1:** Description of variables.

**Variable category**	**Variable name**	**Variable measurement**
Dependent variable	Chronic disease condition	Non-chronic disease = 0; Chronic disease = 1
Grouped variable	Residential address location	In the rural area = 0; In the urban area = 1
Demographic and sociological characteristics	Gender	Women = 0; Men = 1
	Age	60–64 = 1; 65–74 = 2; ≥75 = 3
	Educational attainment	Elementary school and below = 1; Junior school = 2; High School and above = 3
	Marital status	Married with spouses living together = 1; Married with spouses but not living together = 2; Divorced = 3; Widowed = 4; Unmarried = 5
	Minorities	Han = 0; Non-Han = 1
	Religiosities	No = 0; Yes = 1
	Duration of sleep	≤ 4 h = 1; 4–8 h = 2; >8 h = 3 (h = hour)
	Smoking	No = 0; Yes = 1
	Drinking alcohol	No = 0; Yes = 1
	Social activity	No = 0; Yes = 1
	Physical activity	No = 0; Yes = 1
Social system and economic status	Insurance	No = 1; Resident medical insurance# = 2; Urban employee medical insurance = 3; Government medical insurance = 4
	Region	East = 1; Central = 2; West = 3
	Socioeconomic status	Lowest = 1; Less affluent = 2; Average = 3; More affluent = 4; Most affluent = 5

Resident medical insurance# comprises both urban resident medical insurance and new rural cooperative medical insurance.

### 2.4 Statistical analysis

In 1956, the UN categorized people in the ages of 65 years and above in developed nations and 60 years and above in developing nations as older adults (39). Aligning with developing-country norms, older adults were defined as individuals aged 60 years and above in China. This study initially enrolled 10,577 participants aged 60 years and above. After data refinement, excluding non-respondents of chronic disease status (*n* = 40) and unknown respondents (*n* = 53), a total of 10,484 participants remained. First, since there are no equal numbers of participants between rural and urban older adults, it could lead to biases. In this study, the coarsened exact matching (CEM) method was used to match two sets of data on older adults in rural and urban areas to reduce the biases. After matching, rural older adults and urban older adults could become (or very close to) identical in relation to individual characteristics. A total of 5,927 respondents were matched after the CEM method. Rural older adults accounted for 68.64% of the respondents and urban older adults accounted for 31.36%. Second, a description of urban–rural older adults' demographics was conveyed using frequencies and percentages. The chi-square was employed to contrast chronic disease status with categorical variables between urban and rural older adults. Third, odds ratios (OR) of the covariates in logistic regression models were computed to reflect pertinent influences on chronic disease status among rural–urban older adults. Fourth, Fairlie's decomposition was used to analyze the contribution of the differences in suffering from chronic diseases between rural and urban older adults. Fairlie's decomposition aims to decompose health differences into explained and unexplained components. The former is attributed to group variations in visible variables and is typically classified as “endowment.” The second segment elucidates undetected cohort diversity (40–45). Following Fairlie, the non-linear equation can be expressed as follows:


(1) $$\begin{array}{l} Y_{r}=F\left(X_{r}\beta_{r}\right) \end{array}$$

(2) $$\begin{array}{l} Y_{u}=F\left(X_{u}\beta_{u}\right) \end{array}$$

Fairlie's decomposition can be expressed as follows:


(3) $$\begin{array}{l} \bar{{\text{Y}}_{u}}-\bar{{\text{Y}}_{r}}=\left[\overset{N^{u}}{\underset{i=1}{\sum }}\frac{F\left(X_{i}^{u}\beta^{u}\right)}{N^{u}}-\overset{N^{r}}{\underset{i=1}{\sum }}\frac{F\left(X_{i}^{r}\beta^{u}\right)}{N^{r}}\right]+\\ \left[\overset{N^{r}}{\underset{i=1}{\sum }}\frac{F\left(X_{i}^{r}\beta^{u}\right)}{N^{u}}-\overset{N^{r}}{\underset{i=1}{\sum }}\frac{F\left(X_{i}^{r}\beta^{r}\right)}{N^{r}}\right] \end{array}$$

The average chronic disease condition in urban and rural older adults was independently assessed by $\bar{{\text{Y}}_{\text{r}}}$ and $\bar{{\text{Y}}_{\text{u}}}$. *r* and *u* represented rural and urban older adults, encompassing factors such as gender, age, education level, and so on. β represented coefficient. *N*^*r*^ and *N*^*u*^ signified sample sizes for urban and rural older adults, respectively. Statistical analyses were executed with Stata 15.0.

## 3 Results

### 3.1 Characteristics of the participants

Table 2 illustrates the descriptive statistics of rural and urban older adults. After matching, the prevalence of those suffering from chronic diseases was reported to be 1,894 and 953 among rural and urban older adults among 5,927 respondents. From the results, it can be inferred that 48.03% of the older adults had suffered from chronic diseases, while 51.97% had not suffered from chronic diseases. The proportion of men (45.30%) in rural areas was slightly lower than that of women (54.70%), and the proportion of men (48.31%) in urban areas was slightly lower than that of women (51.69%). The proportion of older adults aged 60–64 years in rural areas was 32.84%, the proportion of older adults aged 65–74 years was 51.82%, and the proportion of older adults aged over 75 years was 15.34%, while in urban areas, the proportion was 32.22%, 49.22%, and 18.56%, respectively. In terms of education level, the proportion of rural older adults with elementary school and below education level was 59.98%, the proportion of those with junior school education level was 22.07%, and the proportion of those with high school and above education level was 17.94%, while for urban older adults, the proportion is 29.48%, 21.84%, and 48.68%, respectively. In terms of marital status, the proportion of rural older adults married with spouses living together was 84.44%, the proportion of rural older adults married with spouses but not living together was 0.69%, the proportion of divorced rural older adults was 0.05%, and the proportion of widowed rural older adults was 14.82%, while for urban older adults, the proportion was 82.89%, 1.24%, 0.11%, and 15.76%, respectively. The proportion of older adults in rural areas who were ethnic Han was 98.43%, and the proportion of non-Han was 1.57%, while the proportion of older adults in urban areas was 97.74% and 2.26%, respectively. The proportion of rural older adults with no religious belief was 96.04% and the proportion of those with religious belief was 3.96%, respectively, while the proportion of urban older adults with no religious belief was 94.46% and those with religious belief was 5.54%, respectively. Other characteristics of the participants are all shown in Table 2.

**Table 2:** Descriptive statistics of independent variables.

**Variable**	**Before matching** ***N*** **(%)**		***p*-Value**	**After matching** ***N*** **(%)**		***p*-Value**
	**Rural**	**Urban**		**Rural**	**Urban**	
**Demographic and sociological characteristics**						
Chronic disease condition						
No+	4,222 (53.30)	1,223 (47.70)	< 0.001	2,174 (53.44)	906 (48.74)	< 0.001
Yes	3,699 (46.70)	1,341 (52.30)		1,894 (46.56)	953 (51.26)	
Gender						
Female+	4,022 (50.78)	1,335 (52.07)	0.256	2,225 (54.70)	961 (51.69)	0.053
Male	3,899 (49.22)	1,229 (47.93)		1,843 (45.30)	898 (48.31)	
Age						
60–64+	2,490 (31.44)	828 (32.29)	0.266	1,336 (32.84)	599 (32.22)	0.302
65–74	3,737 (47.18)	1,163 (45.36)		2,108 (51.82)	915 (49.22)	
≥75	1,694 (21.38)	573 (22.35)		624 (15.34)	345 (18.56)	
Education level						
Elementary school and below+	4,937 (62.33)	749 (29.21)	< 0.001	2,440 (59.98)	548 (29.48)	< 0.05
Junior school	1,675 (21.15)	558 (21.76)		898 (22.07)	406 (21.84)	
High School and above	1,309 (16.52)	1,257 (49.03)		730 (17.94)	905 (48.68)	
Marital status						
Married with spouses living together+	5,879 (74.22)	1,942 (75.74)	< 0.001	3,435 (84.44)	1,541 (82.89)	0.101
Married with spouses but not living together	278 (3.51)	82 (3.20)		28 (0.69)	23 (1.24)	
Divorced	75 (0.95)	51 (1.99)		2 (0.05)	2 (0.11)	
Widowed	1,625 (20.52)	481 (18.77)		603 (14.82)	293 (15.76)	
Unmarried	64 (0.80)	8 (0.30)		–	–	
Minorities						
Han+	7,362 (92.94)	2,385 (93.02)	0.896	4,004 (98.43)	1,817 (97.74)	0.064
Non-Han	559 (7.06)	179 (6.98)		64 (1.57)	42 (2.26)	
Religiosities						
No+	7,051 (89.02)	2,300 (89.70)	0.330	3,907 (96.04)	1,756 (94.46)	0.420
Yes	870 (10.98)	264 (10.30)		161 (3.96)	103 (5.54)	
**Life-style and health behavior**						
Duration of sleep						
≤ 4 h+	1,863 (23.53)	416 (16.23)	< 0.001	728 (17.90)	264 (14.20)	0.085
4–8 h	5,040 (63.66)	1,990 (77.64)		3,221 (79.18)	1,528 (82.20)	
>8 h	1,014 (12.81)	157 (6.13)		119 (2.92)	67 (3.60)	
Smoking						
No+	4,342 (54.84)	1,502 (58.63)	< 0.01	2,414 (59.34)	1,086 (58.42)	0.503
Yes	3,575 (45.16)	1,060 (41.37)		1,654 (40.66)	773 (41.58)	
Drinking alcohol						
No+	5,538 (69.95)	1,708 (66.67)	< 0.01	3,001 (73.77)	1,270 (68.32)	0.079
Yes	2,379 (30.05)	854 (33.33)		1,067 (26.23)	589 (31.68)	
Social activity						
No+	4,516 (57.04)	1,017 (39.68)	< 0.001	2,209 (54.30)	748 (40.24)	0.101
Yes	3,401 (42.96)	1,546 (60.32)		1,859 (45.70)	1,111 (59.76)	
Physical activity						
No+	4,610 (58.23)	1,414 (55.17)	< 0.01	2,307 (56.71)	1,008 (54.22)	0.073
Yes	3,307 (42.77)	1,149 (44.83)		1,761 (43.29)	851 (45.78)	
**Social system and economic status**						
Insurance						
No+	273 (3.48)	39 (1.54)	< 0.001	111 (2.75)	27 (1.47)	0.095
Resident medical insurance	7,173 (91.43)	1,154 (45.61)		3,694 (91.39)	852 (46.38)	
Urban employee medical insurance	328 (4.18)	433 (48.74)		199 (4.92)	883 (48.07)	
Government medical insurance	71 (0.91)	104 (4.11)		38 (0.94)	75 (4.08)	
Region						
East+	2,714 (34.59)	675 (27.51)	< 0.001	1,376 (33.94)	536 (29.04)	0.062
Middle	2,619 (33.38)	1,056 (43.03)		1,524 (37.59)	802 (43.45)	
West	2,513 (32.03)	723 (29.46)		1,154 (28.47)	508 (27.52)	
Socioeconomic status						
Lowest+	2,103 (26.59)	372 (14.54)	< 0.001	974 (23.94)	276 (14.85)	0.058
Less affluent	2,086 (26.37)	351 (13.72)		882 (21.68)	259 (13.93)	
Average	1,567 (19.81)	470 (18.37)		859 (21.12)	359 (19.31)	
More affluent	1,359 (17.18)	553 (21.61)		840 (20.65)	423 (22.75)	
Most affluent	795 (10.05)	813 (31.76)		513 (12.61)	542 (29.16)	

+ Reference levels in the regressions; virtual variables for Chi-square test; N (%) were reported.

### 3.2 Comparative analysis of variable distributions in different chronic disease conditions

Table 3 demonstrates the comparative analysis of variable distribution in chronic disease conditions among rural and urban older adults. It was reported that there were significant differences in gender (*p* < 0.05), education level (*p* < 0.001), minorities (*p* < 0.01), religiosities (*p* < 0.01), duration of sleep (*p* < 0.01), smoking (*p* < 0.001), social activities (*p* < 0.001), insurance (*p* < 0.001), and socioeconomic status (*p* < 0.001) between rural and urban older adults suffering from chronic diseases. However, there were significant differences in age (*p* < 0.05), education level (*p* < 0.001), marital status (*p* < 0.05), drinking alcohol (*p* < 0.05), social activity (*p* < 0.001), insurance (*p* < 0.001), region (*p* < 0.001), and socioeconomic status (*p* < 0.001) between rural and urban older adults who had not suffered from chronic diseases.

**Table 3:** Comparative analysis of variable distribution in chronic disease condition.

**Variable**	**Suffering from chronic diseases**			**Not suffering from chronic diseases**		
	**Rural**	**Urban**	***p*** **-Value**	**Rural**	**Urban**	***p*** **-Value**
**Demographic and sociological characteristics**						
Gender						
Female+	1,008 (57.02)	500 (52.47)	< 0.05	1,145 (52.67)	461 (50.88)	0.366
Male	814 (42.98)	453 (47.53)		1,029 (47.33)	445 (49.12)	
Age						
60–64+	572 (30.20)	393 (29.31)	0.301	764 (35.14)	318 (35.10)	< 0.05
65–74	1,014 (53.54)	632 (47.13)		1,094 (50.32)	420 (46.36)	
≥75	308 (16.26)	316 (23.56)		316 (14.54)	168 (18.54)	
Education level						
Elementary school and below+	1,180 (62.30)	297 (31.16)	< 0.001	1,260 (57.96)	251 (27.70)	< 0.001
Junior school	407 (21.49)	208 (21.83)		491 (22.59)	198 (21.85)	
High School and above	307 (16.21)	448 (47.01)		423 (19.46)	457 (50.44)	
Marital status						
Married with spouses living together+	1,559 (82.31)	788 (82.69)	0.465	1,876 (86.29)	753 (83.11)	< 0.05
Married with spouses but not living together	12 (0.63)	8 (0.84)		16 (0.74)	15 (1.66)	
Divorced	–	1 (0.10)		2 (0.09)	1 (0.11)	
Widowed	323 (17.05)	156 (16.37)		280 (12.88)	137 (15.12)	
Minorities						
Han+	1,869 (98.68)	927 (97.27)	< 0.01	2,135 (98.21)	890 (98.23)	0.957
Non-Han	25 (1.32)	26 (2.73)		39 (1.79)	16 (1.77)	
Religiosities						
No+	1,815 (95.83)	891 (93.49)	< 0.01	2,092 (96.23)	865 (95.47)	0.330
Yes	79 (4.17)	62 (6.51)		82 (3.77)	41 (4.53)	
**Life-style and health behavior**						
Duration of sleep						
≤ 4 h+	394 (20.80)	151 (15.84)	< 0.01	334 (15.36)	113 (12.47)	0.079
4–8 h	1,449 (76.50)	770 (80.80)		1,772 (81.51)	758 (83.66)	
>8 h	51 (2.69)	32 (3.36)		68 (3.13)	35 (3.86)	
Smoking						
No+	1,165 (61.51)	570 (59.81)	0.381	1,249 (57.45)	516 (56.95)	0.799
Yes	729 (38.49)	383 (40.19)		925 (42.55)	390 (43.05)	
Drinking alcohol						
No+	1,470 (77.61)	665 (69.78)	< 0.001	1,531 (70.42)	605 (66.78)	< 0.05
Yes	424 (22.39)	288 (30.22)		643 (29.58)	301 (33.22)	
Social activity						
No+	1,003 (52.96)	382 (40.08)	< 0.001	1,206 (55.47)	366 (40.40)	< 0.001
Yes	891 (47.04)	571 (59.92)		968 (44.53)	540 (59.60)	
Physical activity						
No+	1,086 (57.34)	526 (55.19)	0.276	1,221 (56.16)	482 (53.20)	0.132
Yes	808 (42.66)	427 (44.81)		953 (43.84)	424 (46.80)	
**Social system and economic status**						
Insurance						
No+	48 (2.55)	12 (1.28)	< 0.001	63 (2.929)	15 (1.67)	< 0.001
Resident medical insurance	1,717 (91.14)	430 (45.74)		1,977 (91.61)	422 (47.05)	
Urban employee medical insurance	95 (5.04)	452 (48.09)		104 (4.82)	431 (48.05)	
Government medical insurance	24 (1.27)	46 (4.89)		14 (0.65)	29 (3.23)	
Region						
East+	584 (30.97)	275 (29.16)	0.485	792 (36.53)	261 (28.90)	< 0.001
Middle	756 (40.08)	377 (39.98)		768 (35.42)	425 (47.07)	
West	546 (28.95)	291 (30.86)		608 (28.04)	217 (24.03)	
Socioeconomic status						
Lowest+	478 (25.24)	151 (15.84)	< 0.001	496 (22.82)	125 (13.80)	< 0.001
Less affluent	434 (22.91)	140 (14.69)		448 (20.61)	119 (13.13)	
Average	392 (20.70)	178 (18.68)		467 (21.48)	181 (19.98)	
More affluent	377 (19.90)	219 (22.98)		463 (21.30)	204 (22.52)	
Most affluent	213 (11.25)	265 (27.81)		300 (13.80)	277 (30.57)	

+ Reference levels in the regressions; virtual variables for Chi-square test; N (%) were reported.

### 3.3 Associations between chronic disease conditions and their determinants

Table 4 shows the matched logistic results of rural and urban older adults suffering from chronic diseases. For rural older adults, those who were widowers (OR: 1.267, CI: 1.047–1.533), who drink alcohol (OR: 1.421, CI: 1.210–1.668), and having government medical insurance (OR: 4.869, CI: 1.144–1.861) had higher odds of suffering from chronic diseases. However, those who had an education level of high school and above (OR: 0.802, CI: 0.664–0.968), were minorities (OR: 0.608, CI: 0.387–0.955), who reported a duration of sleep of 4–8 h (OR: 0.745, CI: 0.619–0.896) or above 8 h (OR: 0.649, CI: 0.446–0.943), and who had the most affluent socioeconomic status (OR: 0.778, CI: 0.627–0.964) had lower odds of suffering from chronic diseases. As for urban older adults, those who were aged 65–74 years (OR: 1.246, CI: 1.051–1.552) and having government medical insurance (OR: 2.362, CI: 1.203–5.912) had higher odds of suffering from chronic diseases.

**Table 4:** Matched logistic results of rural and urban older adults suffering from chronic diseases.

**Variable**	**Rural**			**Urban**		
	**OR**	**95% CI**		**OR**	**95% CI**	
**Demographic and sociological characteristics**						
Gender	1.141	1.144	1.816	1.212	0.872	1.685
Age						
65–74 years	1.107	0.954	1.284	1.246^*^	1.051	1.552
≥75 years	1.051	0.855	1.292	1.028	0.756	1.397
Education level						
Junior school	0.904	0.765	1.068	0.897	0.682	1.179
High School and above	0.802^*^	0.664	0.968	0.846	0.654	1.094
Marital status						
Married, not cohabiting	0.889	0.487	1.624	0.517	0.204	1.313
Divorced	–	–	–	0.997	0.061	16.42
Widowed	1.267^*^	1.047	1.533	0.990	0.745	1.314
Minorities	0.608	0.387	0.955	1.358	0.708	2.606
Religiosities	1.067	0.807	1.412	1.466	0.960	2.237
**Lifestyle and health behavior**						
Duration of sleep						
4–8	0.745^**^	0.619	0.896	0.788	0.598	1.039
>8	0.649^*^	0.446	0.943	0.700	0.402	1.217
Smoking	1.200	0.973	1.481	1.145	0.839	1.564
Drinking alcohol	1.421^***^	1.210	1.668	1.119	0.884	1.416
Social activity	0.778^***^	1.122	1.473	0.902	0.893	1.344
Physical activity	1.011	0.887	1.153	0.928	0.763	1.128
**Social system and economic status**						
Insurance						
Resident medical insurance	1.318	0.873	1.989	1.268	0.576	2.793
Urban employee medical insurance	1.914	1.187	3.085	1.426	0.643	3.162
Government medical insurance	4.869^***^	2.407	9.851	2.352^*^	1.203	5.912
Region						
Central	1.062	0.910	1.239	0.835	0.665	1.049
West	1.031	0.866	1.227	1.266	0.978	1.639
Socioeconomic status						
Less affluent	0.989	0.782	1.251	1.013	0.713	1.440
Average	0.888	0.712	1.107	0.918	0.661	1.274
More affluent	0.872	0.703	1.080	0.969	0.703	1.336
Most affluent	0.778^*^	0.627	0.964	0.880	0.638	1.215
Constant	0.437^**^	0.254	0.752	0.748	0.283	1.982
Prob > chi^2^	< 0.001			< 0.01		
Number of observation	4,026			1,824		

* p < 0.05.

** p < 0.01.

*** p < 0.001.

All predictors entered the logistic regression simultaneously.

### 3.4 Decomposition analysis

This study aimed to explore the differences in suffering from chronic diseases between rural and urban older adults. The health differences between the two groups are shown in Table 5. Moreover, 23.57% of the differences in chronic disease conditions were influenced by considered factors, while 76.43% could be attributed to urban and rural areas. This study showed that duration of sleep (−6.60%), drinking alcohol (−10.80%), social activity (14.77%), and region (6.95%) significantly influenced chronic disease conditions.

**Table 5:** Fairlie's decomposition of differences in suffering from chronic diseases between rural and urban older adults.

**Decomposition terms**	**Chronic disease condition**		
Difference	−0.0441		
Explained (%)	−0.0104 (23.57%)		
**Explained**			
**Contribution to difference**	**Contribution (%)**	**95% CIs**	
Gender	2.83%	−0.0036	0.0011
Age	−0.16%	−0.0015	0.0016
Education level	−27.17%	−0.0024	0.0264
Marital status	0.96%	−0.0015	0.0007
Minorities	−0.91%	−0.0003	0.0011
Religiosities	−0.19%	−0.0012	0.0013
Duration of sleep	−6.60%^**^	0.0013	0.0046
Smoking	−0.41%	−0.0009	0.0013
Drinking alcohol	−10.80%^**^	0.0023	0.0072
Social activity	14.77%^**^	−0.0109	−0.0022
Physical activity	−0.09%	−0.0008	0.0009
Insurance	57.95%	−0.0582	0.0070
Region	6.95%^**^	−0.0052	−0.0010
Socioeconomic status	−13.77%	−0.0031	0.0152

^*^p < 0.05.

^*^p < 0.01.

^***^p < 0.001.

95% CI, 95% coefficient interval.

## 4 Discussion

### 4.1 Summary of the main findings

This study conducted a comparative analysis in China examining the differences in suffering from chronic diseases between rural and urban older adults (aged 60 years and above) and the nation with the largest older adult population. This study provided empirical data evaluating health differences among rural and urban older adults.

This study indicated that 48.03% of older adults were grappling with chronic diseases, with a notably increased proportion in urban areas (51.26%) compared to rural areas (46.56%). The finding was similar to *An Analysis Report of the Sixth National Health Services Survey in China* (46), which reported that 59.10% of older adults were suffering from chronic diseases, and the proportion of urban older adults (60.60%) suffering from chronic diseases was higher than that of rural older adults (57.50%), which might be because the urban older adults had a high level of education, a strong awareness of prevention and healthcare, and go to hospitals with a high level of medical care after being ill. On the contrary, the rural older adults had a weak awareness of self-care due to factors such as economic status and medical conditions, and some older adults had not had a physical examination for several years or even decades and had never gone to a doctor without serious clinical symptoms, which led to a high prevalence of older adults suffering from chronic diseases and a low rate of them seeing a doctor in rural areas (47). In 2017, the Chinese government launched the *China medium and long-term plan for the prevention and treatment of chronic diseases (2017–2025)*, which called for targeted prevention and control measures based on the epidemic characteristics of chronic diseases in different areas and different population groups (48). Therefore, the construction of rural healthcare should strengthen the prevention and treatment of older adults suffering from chronic diseases.

Many studies have shown that chronic disease prevalence increased significantly with age (23, 25), and the logistic regression results showed that participants aged 65–74 years in urban areas projected a heightened prevalence of chronic diseases. This was largely attributed to the escalating risk of hypertension, diabetes, coronary heart disease, and other chronic diseases with age (25). For example, studies by Brustio et al. (49) and Béghin et al. (50) indicated that the physical function of older adults would decline with age. Therefore, age is an important consideration in the prevention and treatment of chronic diseases. Compared to the education level of elementary school and below, the rural older adults with an education level of high school and above had a lower prevalence of chronic diseases, which might be because older adults with a higher education level would have a better chance of learning about health and possessing better health literacy. Furthermore, relevant studies showed that chronic patients with higher education had better self-efficacy (51). This study showed a higher prevalence of chronic diseases among widowed rural older adults. The reason might be that the physical health of older adults in rural areas deteriorates when they are helpless because of a lack of companionship and care of their spouses. This finding was similar to the finding in England by Nartey et al. (52), which showed that chronic diseases were more prevalent among widowed or divorced individuals. This study showed that older adults who drink alcohol had a higher prevalence of chronic diseases in rural areas, which was similar to some studies that indicated that drinking alcohol could lead to a variety of diseases. A number of studies have shown that drinking alcohol is associated with many chronic diseases (53, 54). For example, drinking alcohol can cause damage to the heart and heart muscle cells, which can lead to heart failure. Even worse, long-term drinking of alcohol can cause osteoporosis, bone pain, and fracture. The harm of drinking alcohol to the human body is various (55). Acetaldehyde, a non-oxidative metabolite of alcohol, has been identified as a kind of carcinogen by the cancer research organization of the World Health Organization, including oral cancer, laryngeal cancer, esophageal cancer, liver cancer, colorectal cancer, and so on (56). In a word, drinking alcohol can increase the risk of suffering from chronic diseases among older adults as their physical functions deteriorate. This study showed a lower prevalence of chronic diseases among rural older adults who participated in social activities. The reason might be that participating in social activities could bring emotional value and promote health. This finding was similar to the study by Jeon and Park (57), which reported participating in social activities could improve the health of older adults in Korea. This study showed that older adults with government medical insurance had a higher prevalence of chronic diseases both in rural and urban areas. The result was similar to the study by Zhou et al. (58), which reported that the prevalence of chronic diseases was higher among older adults with government medical insurance and was obviously higher than the older adults with urban employee medical insurance and resident medical insurance. In addition, older adults with urban employee medical insurance had a higher prevalence of chronic diseases in urban areas, which was mainly due to the difference in the guaranteed ability of different health insurance. In China, the security ability of government medical insurance, urban employee medical insurance, resident medical insurance, and no insurance ranked from high to low (59). Kaliski (60) discovered that health insurance could increase the consumption of healthcare resources. For example, Niu et al. (54) found that the out-patient and in-patient services of the residents with urban employee medical insurance were 2.7 times and 1.80 times of the residents with resident medical insurance, respectively. Therefore, in the process of exploring the integration of urban and rural residents' health insurance, the government should pay attention to reducing the difference in medical service utilization among different health insurance (61). This study showed that rural older adults with a duration of sleep of 4–8 h and above had a lower prevalence of chronic diseases in rural and urban areas. The reason might be the crucial role of appropriate rest in optimizing bodily functions and fostering physiological wellbeing among older adults, typically frailer than before (38). A study by Laposky et al. (62) revealed diminished sleep health conditions, encompassing inadequate sleep duration, diverse sleep timings, low sleep quality, and sleep circadian disruptions, which were prevalent in contemporary societies and linked with multiple disease risks and consequences, including those related to health disparities (e.g., cardiovascular disease, obesity and diabetes, mental disorders, and cancer). Therefore, keeping adequate sleep time is very important for the health of older adults. This study showed that the rural older adults with the most affluent socioeconomic status had a lower prevalence of chronic diseases. The explanation might be that wealthy older adults had better nutrition and access to health services. This finding was similar to the study in Canada by Shah (63), which showed that chronic diseases were more prevalent among individuals from lower socioeconomic status backgrounds.

In addition, Fairlie's decomposition results showed that duration of sleep, drinking alcohol, social activity, and region were the factors of urban–rural differences in chronic disease conditions among older adults in China. The reason might be that there was a dualistic urban–rural structure. In other words, the level of urban and rural economic development, living environment, and medical services are not balanced in China (64). Therefore, this study indicated compellingly that the government should pursue strategies to narrow the urban–rural gap.

### 4.2 Implications

Based on the urban–rural differences in chronic disease conditions, this study has some implications. First, as for duration of sleep, older adults should pay attention to the quality of sleep, and sleep disorders should be treated in time (65). Second, as for drinking alcohol, the government should strengthen the promotion of health literacy among older adults, especially to reduce alcohol consumption. Third, this study showed that social activity had a positive effect on the health of older adults. At present, community home-based older adult care is the most dominant model in China (66). Therefore, the role of social activity in home-based older adult care should be paid attention to. Fourth, as for region, the government should further strengthen health support, such as free medical check-ups and free vaccinations for older adults, to reduce the health differences among older adults in different areas in China.

### 4.3 Limitations

Notably, there are several limitations in this research. First, given the cross-sectional nature of CHARLS (wave 4), tracking shifts at distinct intervals might pose challenges. Second, chronic diseases were classified as suffering from chronic diseases and not suffering from chronic diseases, overlooking the intensity of suffering from chronic diseases. Third, despite numerous factors linked to chronic diseases of older adults, further analysis was restricted. Despite these constraints, this study might illuminate the differences in chronic disease conditions between rural and urban older adults in China. In future research, more complete data and other influencing factors will be considered to validate the findings of this study.

## 5 Conclusion

This study focused on the differences in chronic disease conditions between rural and urban older adults in China. The proportion of urban older adults was higher than that of rural older adults suffering from chronic diseases. Factors including drinking alcohol, social activity, and assets should be addressed to reduce the urban–rural older adults differences in chronic disease conditions in China. This study may help provide evidence for health policies in reducing urban–rural older adults' differences and breaking down the dual barriers in urban and rural areas in an aging society in China. At the same time, this study provided evidence for future research on the health of older adults.

## Data availability statement

Publicly available datasets were analyzed in this study. This data can be found at: http://charls.pku.edu.cn/en.

## Author contributions

JZ: Conceptualization, Data curation, Formal analysis, Funding acquisition, Writing – original draft. YZ: Funding acquisition, Project administration, Writing – review & editing.
