# Supplementary material for: Decomposing differences in the chronic disease condition between rural and urban older adults in China: a cross-sectional analysis
Source: Front Public Health. 2024 Jan 5;11:1298657. doi: 10.3389/fpubh.2023.1298657 (PMC10797097; doi:10.3389/fpubh.2023.1298657)
Supplement: Supplementary file 1 [file Data_Sheet_1.docx]

**Supplementary questionnaire**

In the CHARLS questionnaire, the status of chronic diseases could be reflected as the following questions:

1. Have you been diagnosed with Hypertension by a doctor?
2. Have you been diagnosed with Dyslipidemia (elevation of low density lipoprotein, triglycerides (TGs), and total cholesterol, or a low high density lipoprotein level) by a doctor?
3. Have you been diagnosed with Diabetes or high blood sugar by a doctor?
4. Have you been diagnosed with Cancer or malignant tumor (excluding minor skin cancers) by a doctor?
5. Have you been diagnosed with Chronic lung diseases, such as chronic bronchitis ,emphysema ( excluding tumors, or cancer) by a doctor?
6. Have you been diagnosed with Liver disease (except fatty liver, tumors, and cancer) by a doctor?
7. Have you been diagnosed with Heart attack, coronary heart disease, angina, congestive heart failure, or other heart problems by a doctor?
8. Have you been diagnosed with Stroke by a doctor?
9. Have you been diagnosed with Kidney disease (except for tumor or cancer) by a doctor?
10. Have you been diagnosed with Stomach or other digestive diseases (except for tumor or cancer) by a doctor?
11. Have you been diagnosed with Emotional, nervous, or psychiatric problems by a doctor?
12. Have you been diagnosed with Memory-related disease (such as dementia, brain atrophy, and Parkinson’s disease) by a doctor?
13. Have you been diagnosed with Arthritis or rheumatism by a doctor?
14. Have you been diagnosed with Asthma by a doctor?
